# Cardiac Safety of Diclofenac at a Single Dose in Ram

**DOI:** 10.1155/2013/808731

**Published:** 2013-10-09

**Authors:** Ayse Er, Burak Dik, Orhan Corum, Gul Cetin

**Affiliations:** ^1^Department of Pharmacology and Toxicology, Faculty of Veterinary Medicine, University of Selcuk, 42075 Konya, Turkey; ^2^Department of Pharmacology and Toxicology, Faculty of Veterinary Medicine, University of Dicle, 21280 Diyarbakir, Turkey; ^3^Department of Pharmacology and Toxicology, Faculty of Veterinary Medicine, University of Mehmet Akif Ersoy, 15030 Burdur, Turkey

## Abstract

Nonsteroidal anti-inflammatory drugs are frequently prescribed drug group in human and veterinary medicine. However, diclofenac, a traditional nonsteroidal anti-inflammatory drug, related to cardiotoxicity is reported, and blood cardiac damage markers may increase within the first hours after damage. The aim of the current research was to determine the effect of diclofenac on the blood cardiac damage markers. Single dose of diclofenac (2.5 mg/kg, IM) was injected to 6 rams. Blood samples were collected in before (0 hour, control) and 6 hours after injection. Specific (troponin I, and creatine kinase-MB) and nonspecific (lactate dehydrogenase, aspartate aminotransferase) blood cardiac damage marker concentrations, routine biochemical (hepatic damage, renal damage, lipid metabolism, glucose, and phosphorus) parameters, and hemogram values were measured. Diclofenac increased (*P* < 0.05) specific (troponin I) and nonspecific cardiac (lactate dehydrogenase, aspartate aminotransferase), hepatic (aspartate aminotransferase, alkaline phosphatase, and alanine aminotransferase), and muscular (creatine kinase) damage markers and high density lipoprotein level, while it decreased (*P* < 0.05) low density lipoprotein level. Moreover, diclofenac decreased (*P* < 0.05) white blood cell counts and increased (*P* < 0.05) red blood cell counts. In conclusion, it may be stated that diclofenac shows slight cardiotoxicity, whereas it may show potent hepatic and muscular damage effects at an intramuscularly single dose in sheep. Thereby, repeated injections of diclofenac may be more harmful in sheep.

## 1. Introduction

Nonsteroidal anti-inflammatory drugs (NSAIDs) are most prescribed drugs in human and veterinary medicine that provide anti-inflammatory, antipyretic, analgesic, antispasmodic, and anticoagulant effects. Diclofenac (2-(2,6-dichloranilino) phenylacetic acid), a phenylacetic acid derivative NSAID, is one of the most frequently prescribed nonselective NSAIDs worldwide, and it has strong analgesic, antipyretic, and anti-inflammatory effects. It is believed that diclofenac shows its action via inhibition of prostaglandin synthesis by inhibiting cyclooxygenase (COX) and lipoxygenase enzyme pathway. Intravenous, intramuscular, oral, suppository, transdermal patch, and gel forms of diclofenac are available in markets for human and veterinary medicine. It is commonly used to treat bone-muscle traumas, osteoarthritis, rheumatoid arthritis, ankylosing spondylitis, colic, and infectious hyperthermia [1–5].

Most frequently used NSAIDs worldwide have serious side effects, such as death. It has been reported that the application of the drugs in this group may be risky for cardiac and newborn infant patients as well as healthy individuals [4–6]. Moreover, it has been stated that treatment with NSAIDs has serious side effects such as gastrointestinal ulceration or bleeding, liver and kidney damage, allergic reactions, myocardial infarction, and cardiac sudden death [3, 7–10]. It has been indicated that the application of diclofenac, a nonselective NSAID, may cause cardiovascular problems and increase myocardial infarction risk or may be more risky for patients with coronary heart disease and myocardial infarction [4, 6, 10, 11]. However, it has been postulated that parameters detected in blood as a marker of cardiac damage (troponin I (TI), creatine kinase-MB (CK-MB), lactate dehydrogenase (LDH), aspartate aminotransferase (AST)) may increase in the first 4–6 hours following the damage and this approach may be estimated for cardiac damage in chemically-induced cardiotoxicity researches in the first hours of blood sampling [12–16]. 

EMEA approves that the dose of diclofenac in cattle and porcine is 2.5 mg/kg/day, intramuscularly for 1–3 days. However, the extra label use of diclofenac is also available in sheep and other animal species. In a research in sheep, it has been reported that 1.4 mg/kg diclofenac may be used intramuscularly every six hours [5, 17]. 

Potential cardiac side effects, frequent usage of extra label, and limited three-day usage procedure of diclofenac [2, 3, 8–10, 17] were taken into consideration; it was hypothesized that singly dose of diclofenac might increase blood cardiac damage markers in rams.

The aim of the present study was to determine the effect of intramuscular injection of 2.5 mg/kg diclofenac on the markers of blood cardiac (TI, CK-MB, LDH, and AST), hepatic (alkaline phosphatase (ALP), alanine aminotransferase (ALT), AST, gamma glutamyltransferase (GGT), total protein (TP), albumin), renal (creatinine, blood urea nitrogen (BUN)) and muscle (creatine kinase (CK)) damage markers as well as lipid metabolism products (cholesterol, triglyceride, high density lipoprotein (HDL), low density lipoprotein (LDL)), blood cell counts (white blood cells counts (WBC), red blood cell counts (RBC), thrombocyte counts, hematocrit, and hemoglobin) and other biochemical parameters (glucose, and phosphorus). 

## 2. Material and Methods

In the current study, six crossbred merino rams (1-2 years old, 60–80 kg) were used. All experimental procedures were approved by Ethical Committee of Faculty of Veterinary Medicine, Selcuk University (no. 2013/13). Rams were administered with intramuscular (IM) single dose (2.5 mg/kg) diclofenac (Dikloron amp. 75 mg/mL, Deva, Istanbul, Turkey). Blood samples were collected from *V. jugularis* before (0 hour, control) and six hours after administration and centrifuged to obtain blood serum and plasma samples. The concentrations of serum TI (Siemens Advia Centaur CP, USA), plasma CK-MB, LDH, AST, ALP, ALT, GGT, TP, albumin, creatinine, BUN, CK, cholesterol, triglyceride, HDL, LDL, glucose, and phosphorus were analyzed by autoanalyzer (ILab-300 bioMerieux Diagnostics, Milan, Italy). Hemogram values (WBC, RBC, thrombocyte, hematocrit, and hemoglobin) were measured by blood cell counter (Shenzhen mindray Bio-Medical Electronics, BC-2800 Auto Hematology Analyzer, China).

The data were analyzed by paired *t*-test (SPSS 19.0 for Windows) and all values were presented as mean ± SEM. The differences were considered significant at *P* < 0.05.

## 3. Results

The concentrations of cardiac damage markers are given in Table 1, whereas the concentrations of hepatic, renal, and muscle damage markers, lipid metabolism products and other biochemical parameters are shown in Table 2. Diclofenac increased (*P* < 0.05) the concentrations of TI, LDH, and AST detected in the 6th hour as compared to 0 time (control). Moreover, the concentrations of ALP, ALT, CK, and HDL in the 6th hour were higher (*P* < 0.05) than control, whereas the concentration of LDL detected in the 6th hour was lower (*P* < 0.05). In addition, diclofenac decreased (*P* < 0.05) WBC, while it increased (*P* < 0.05) RBC compared to control (Table 3).

## 4. Discussion

It is well known that NSAIDs which have extensive usage in human and veterinary medicine might cause life-threatening side effects such as myocardiac infarction and cardiac sudden death. Diclofenac is one of the most used nonselective NSAIDs due to its strong analgesic, antipyretic, and anti-inflammatory effects [3–6, 9]. It has been indicated that the rate of removing the drugs from market is approximately 9% due to drug-induced cardiotoxicity [18].

In the present study, diclofenac increased the concentrations of TI (*P* < 0.030), CK-MB (*P* < 0.080), LDH (*P* < 0.018), and AST (*P* < 0.038) at the rates of 125%, 22.1%, 23.9% and 87.8%, respectively (Table 1). The concentrations of TI which plays a role in the regulation of contractility of heart muscle and CK-MB which is the subtype of creatine kinase that plays a role in cell energy metabolism are considered as specific markers for acute cardiac damage, whereas LDH and AST are traditionally predicated on nonspecific cardiac damage markers; thereby they are evaluated together with specific markers [14, 15, 18–20]. Concentrations of cardiac damage markers in blood may increase in the first six hours in experimentally drug-induced cardiotoxicity [12, 13, 16]. Higher concentrations of CK-MB and TI were reported in a patient who used diclofenac and had acute myocardial infarction [21]. It has been stated that cardiac damage originating from NSAIDs might be related to the inhibition of COX synthesis by this group of drugs. There are two types of COX enzymes: COX-1, which is responsible for vasoconstriction, and stimulation of platelet aggregation, and COX-2, which is usually specific to inflamed tissue and causes vasodilatation by inhibiting synthesis of prostacyclin and inhibition of platelet aggregation. The reason of cardiac side effects of NSAIDs has been shown to form imbalance between COX-1 and COX-2. Therefore, specific COX-2 inhibitor NSAIDs have been removed from market due to cardiac side effects such as myocardial infarction and cardiac sudden death. It has been reported that diclofenac has a high inhibitory effect on COX-2 rather than COX-1 and has similar cardiac side effects as selective COX-2 inhibitory NSAIDs [3, 7, 9–11]. Diclofenac caused increases in the cardiac damage markers may be related to inhibitory effect of COX-2 than COX-1 and/or ram may be more susceptible to drug than cow. 

In the present study, diclofenac increased (*P* < 0.05) the concentrations of hepatocellular damage markers ALT, AST which played role in amino acid metabolism and ALP which existed intensively in bile duct (Table 2). ALT is a more remarkable marker for hepatic damage than AST that exists also in heart muscle [22]. Damaging effect of NSAIDs on liver is well known. Moreover, it has been reported that diclofenac increases transaminases (ALT, and AST) rather than NSAIDs; therefore, measurement of transaminases may be beneficial, during diclofenac treatment [3, 5, 23]. 

In the current study, diclofenac treatment did not alter (*P* > 0.05) the concentrations of BUN and creatinine, renal function test (Table 2). Although short-term diclofenac treatment does not cause any complications, long-term diclofenac treatment may be monitored for renal damage [3].

Intramuscular injection of diclofenac increased (*P* < 0.008) the plasma concentration of CK (Table 2). CK concentration in blood is mainly originated from muscle damage [24], and increased CK concentration in the current study may be derived from muscle damage during diclofenac injection. It was determined that diclofenac treatment increased (*P* < 0.011) the concentration of plasma HDL, while it decreased (*P* < 0.002) the concentration of LDL (Table 2). Similar results were reported following aspirin, naproxen, and ibuprophene treatments [25–27]. Paradoxically, diclofenac caused decrement (*P* < 0.006) in WBC, whereas it increased (*P* < 0.019) RBC in this study (Table 3). Nevertheless, this effect of diclofenac might not be explained.

In conclusion, it is suggested that diclofenac, recommended at the dose of 2.5 mg/kg/day for 1–3 days in cattle and porcine and also used in other species without approvement, may cause cardiac and hepatic damage; thereby, more harmful damages by repeated injections may occur in untargeted species, such as sheep.

## Figures and Tables

**Table 1:** The effect of diclofenac (2.5 mg/kg, IM) treatment on cardiac damage markers in rams (mean ± SEM).

Parameters	0 (control) hour	6 hours	% alteration	*P* values
TI (ng/mL)	0.04 ± 0.01	0.09 ± 0.01	+125	*P* < 0.030
CK-MB (U/L)	131 ± 6.42	160 ± 11.9	+22.1	*P* < 0.080
LDH (U/L)	978 ± 64.2	1212 ± 107	+23.9	*P* < 0.018
AST (U/L)	54.3 ± 4.72	102 ± 17.5	+87.8	*P* < 0.038

TI: troponin I, CK-MB: creatine kinase-MB, LDH: lactate dehydrogenase, and AST: aspartate aminotransferase.

**Table 2:** The effect of diclofenac (2.5 mg/kg, IM) treatment on the plasma concentrations of hepatic, renal, and muscular damage markers; lipid metabolism products; some biochemical parameters in rams (mean ± SEM).

Parameters	0 (control) hour	6 hour	% alteration	*P* values
ALP (U/L)	26.6 ± 4.00	73.5 ± 14.0	+176	*P* < 0.016
ALT (U/L)	13.3 ± 1.35	19.0 ± 2.48	+42.9	*P* < 0.038
GGT (U/L)	56.3 ± 4.61	53.0 ± 5.23	−5.86	*P* > 0.05
TP (g/dL)	6.73 ± 0.17	6.76 ± 0.16	+0.45	*P* > 0.05
Albumin (g/dL)	3.35 ± 0.17	3.33 ± 0.06	−0.60	*P* > 0.05
Creatinine (mg/dL)	0.83 ± 0.07	0.89 ± 0.10	+7.23	*P* > 0.05
BUN (mg/dL)	14.0 ± 1.75	15.5 ± 2.14	+10.7	*P* > 0.05
CK (U/L)	100 ± 13.5	1690 ± 369	+1590	*P* < 0.008
Cholesterol (mg/dL)	226 ± 6.58	227 ± 8.58	+0.44	*P* > 0.05
Triglyceride (mg/dL)	23.8 ± 1.75	29.8 ± 2.78	+25.2	*P* > 0.05
HDL (mg/dL)	30.8 ± 4.40	36.3 ± 4.49	+17.9	*P* < 0.011
LDL (mg/dL)	15.3 ± 2.15	13.5 ± 1.87	−11.8	*P* < 0.002
Glucose (mg/dL)	72.6 ± 5.29	90.3 ± 16.1	+24.4	*P* > 0.05
Phosphorus (mg/dL)	5.66 ± 0.35	5.73 ± 0.48	+1.24	*P* > 0.05

ALP: alkaline phosphatase, ALT: alanine aminotransferase, GGT: gamma glutamyltransferase, TP: total protein, CK: creatine kinase, BUN: blood urea nitrogen, HDL: high density lipoprotein, and LDL: low density lipoprotein.

**Table 3:** The effect of diclofenac (2.5 mg/kg, IM) on hemogram parameters in rams (mean ± SEM).

Parameters	0 (control) hour	6 hours	% alteration	*P* values
WBC (×10^9^/L)	7.29 ± 1.14	5.81 ± 0.98	−20.30	*P* < 0.006
Lymphocyte %	64.7 ± 2.28	53.2 ± 4.14	−17.8	*P* < 0.041
Monocyte %	3.46 ± 0.46	3.30 ± 0.32	−4.62	*P* > 0.05
Granulocyte %	31.8 ± 1.99	42.5 ± 3.98	+33.6	*P* < 0.032
RBC (×10^12^/L)	13.0 ± 0.28	14.0 ± 0.26	+7.69	*P* < 0.019
Thrombocyte (×10^9^/L)	355 ± 74.0	436 ± 44.9	+22.8	*P* > 0.05
Hematocrit %	36.8 ± 1.03	38.7 ± 1.70	+5.16	*P* > 0.05
Hemoglobin (g/L)	11.7 ± 0.33	12.2 ± 0.35	+4.27	*P* > 0.05

WBC: white blood cell; RBC: red blood cell.
